# Reusable gelatin-based inks for 3D printing of veterinary gabapentin tablets: a sustainable approach

**DOI:** 10.3389/fvets.2026.1835903

**Published:** 2026-05-13

**Authors:** Alex Taylor, Jarkko Ketolainen, Mattias Paulsson, Jonas Autenrieth, Jonas Lindh, Henrik Rönnberg

**Affiliations:** 1 School of Pharmacy, University of Eastern Finland, Kuopio, Finland; 2 Department of Women’s and Children’s Health, Uppsala University, Uppsala, Sweden; 3 Division of Molecular Pharmaceutics, Department of Pharmacy, Uppsala Biomedical Center, Uppsala University, Uppsala, Sweden; 4 Division of Nanotechnology and Functional Materials, Department of Materials Science and Engineering, Uppsala University, Uppsala, Sweden; 5 Department of Animal Biosciences, Swedish University of Agricultural Sciences, Uppsala, Sweden

**Keywords:** 3D printing, gabapentin, reusability, semi-solid extrusion, sustainability, veterinary medicine

## Abstract

Gabapentin (GAB), an anticonvulsant, is commonly used in veterinary medicine to manage anxiety, pain, and epilepsy. Although it is widely used, achieving personalized dosing for small animals remains a significant challenge. Three-dimensional printing (3DP), particularly semi-solid extrusion (SSE), has shown promise in producing individualized dosage forms efficiently and with precision. Despite its advantages, SSE is limited by the need to freshly prepare the ink for printing and the extended post-processing times required for curing or solidifying the printed objects. This study investigated for the first time the feasibility of reusing a formulation designed for veterinary patients. The chewable tablets in this study were made using an already available proprietary gelatin-based excipient base, CuraVet®. Two formulations with varying GAB concentrations were printed using the same syringe initially and then again after 14 days of refrigerated storage. The formulation that contained a lower concentration of GAB maintained printability and dosing precision without requiring adjustments to printer settings, enabling the production of chewable tablets in under 30 min with a minimal amount of active work required. In contrast, the higher concentration formulation exhibited crystal formation, indicating instability. This proof-of-concept study demonstrated the feasibility of using reusable gelatin-based inks for on-demand 3D printing of veterinary medicines, highlighting their potential for clinical adoption in veterinary practices and pharmacies, provided that stability is carefully characterized.

## 1 Introduction

Gabapentin (GAB), a structural analogue of gamma-aminobutyric acid, is widely used in veterinary medicine for managing chronic neuropathic pain, anxiety, and as an adjunctive treatment for seizures in companion animals (1). However, its use in veterinary settings is complicated due to the lack of species-specific formulations. This leads to circumstances where GAB is often administered as human-registered capsules or tablets, which are not designed for veterinary patients, for whom palatability (taste, shape, and color) plays a crucial role (2). It is also worth noting that since the required dose varies not only between species but also with the individual animal’s weight and clinical condition, these products are frequently split to try to achieve the needed dose, which has been shown to lead to dose variability even when commercial veterinary products are used (1, 3). These limitations highlight the need for flexible, individualized dosage forms tailored to the specific needs of veterinary patients.

Three-dimensional printing (3DP) has emerged as a promising technology to address these challenges by enabling the production of personalized dosage forms with precise drug loading and customizable shapes (4, 5). Among the various 3D printing techniques, semi-solid extrusion (SSE), also known as pressure-assisted microsyringe printing, has gained attention for its ability to process heat-sensitive drugs at low temperatures and to produce chewable, flavored tablets suitable for diverse patient groups, including pediatric and veterinary populations (6, 7). Since the technique uses a gel or paste that has a certain concentration of the API, it can be used to produce multiple different doses to tailor to the needs of individual patients, such as dogs that need dose adjustments for chronic neuropathic pain (1). Despite its advantages, SSE still faces practical limitations, such as the need to freshly prepare inks for each print and the single-use nature of the systems, which often generates waste (8). The post-processing times can also be as long as 12 h and require the use of additional equipment, such as dryers, to obtain a final product (9).

To improve the sustainability and clinical applicability of SSE, recent studies have explored the use of reusable systems and ink formulations that could be stored and reused without compromising print quality (8, 10). These studies have been promising, but it has been found that maintaining consistent printability and drug content over time remains a challenge, since inks may undergo physical or chemical changes during storage. The main physical changes have been the noticeable increase in yield stress, which leads to the need to change the printing parameters to obtain the same results, and the possibility of phase separation during storage (10, 11). Chemical changes have included modifications of the color and a possible decrease in the drug content due to degradation (8). These types of changes pose a risk to product quality and would need further pharmacopeial standardization or rigorous testing to ensure that the final product is always usable after storage.

This study aimed to evaluate the feasibility of using gelatin-based ink for 3D printing chewable GAB tablets tailored to individual animal weights, using SSE into blister molds, which define the geometry of the final dosage form; this differs from layer-by-layer deposition but retains the key feature of digitally controlled extrusion. It assessed whether tablets could be accurately printed using the same syringe after 14 days of refrigerated storage, without changing the printing parameters. The tablets were analyzed for GAB content using high-performance liquid chromatography (HPLC) and tested for physical and chemical stability of the active pharmaceutical ingredient (API). Although reusability of 3D printing inks has been explored in human pharmaceutical research, veterinary applications have received little attention. This is the first study to investigate ink reusability specifically for veterinary formulations. Reusability supports sustainable manufacturing, enables efficient small-batch production, and helps reduce material waste.

Beyond the environmental advantages, reusable ink systems may also improve clinical practice by streamlining compounding workflows, reducing the workload for pharmacists and veterinarians, and lowering costs. Since pharmaceutical mixers often require a minimum volume of material, being able to reuse the same syringe ensures that leftover ink is not discarded unnecessarily, making the process more practical for individualized veterinary dosing. Importantly, individualized 3D-printed chewable tablets may reduce the stress associated with tablet splitting or forced administration, thereby improving owner compliance and animal welfare. In chronic conditions such as neuropathic pain, where frequent dose adjustments are needed, reusable ink systems have the potential to provide more reliable, palatable, and patient-friendly treatments directly at the point of care.

## 2 Materials and methods

### 2.1 Materials

GAB and Polysorbate 80 (PS80) were obtained from Magis Pharma (Antwerpen, Belgium). Technical grade Curavet® and 100 mL syringes were sourced from CurifyLabs (Helsinki, Finland). 3/16″ Mini Medi-Cup Blisters and LaserLabel™ “25” Lid-Label® Cover Sheets (Medi-Dose Inc., Ivyland, PA, USA) and Gako HV + LV mixing jars (Gako International GmbH, Munich, Germany) were also purchased from CurifyLabs. Analytical-grade monobasic potassium phosphate was acquired from Sigma-Aldrich (St. Louis, MO, USA).

HPLC-grade acetonitrile, water, and analytical-grade potassium hydroxide were purchased from VWR International AB (Stockholm, Sweden). Nylon filters with a 0.45 μm pore size and HPLC vials with 0.45 μm polytetrafluoroethylene filters were obtained from Cytiva (Marlborough, MA, USA). Aluminum crucibles and lids were sourced from DSC Consumables (Austin, MN, USA).

### 2.2 Ink preparation

To evaluate the reusability of pharmaceutical ink formulations after cold storage, both placebo and GAB-loaded inks were prepared using technical grade Curavet®, a proprietary ink material composed of three main components: water, microcrystalline cellulose, and gelatin (12). PS80 was also added as a surfactant at a concentration of 1% (w/w). For the placebo formulation, two 20 g batches were prepared by weighing the appropriate amounts of Curavet® and PS80 into Gako HV + LV mixing jars using an A&D HA-180 analytical balance (A&D Company, Tokyo, Japan). The mixtures were homogenized for 7 min using a Gako PM140 planetary mixer (Gako International GmbH, Munich, Germany). After mixing, the formulations were transferred into 100 mL plastic syringes and stored at 3–4 °C until further use.

For the actual experiment, six GAB-loaded batches were prepared: three batches with 5% (w/w) GAB (Formulation I, 30 g each) and three with 10% (w/w) GAB (Formulation II, 50 g each). One additional 50 g placebo batch was also prepared as a control. Each batch was prepared by weighing GAB, PS80, and Curavet® into a mixing jar using the same analytical balance as before. The components were mixed for 7 min in the planetary mixer. The resulting formulations were then transferred into 100 mL syringes, used once to print, and afterwards stored at 3–4 °C until further use. To keep them in the same position during storage, all syringes containing GAB were stored at a 45-degree angle using a custom 3D-printed holder, while the placebo syringe was stored horizontally.

### 2.3 Initial testing and SSE 3D printing

For the initial printability assessment of the placebo batches, two syringes were removed from cold storage after 4 days and heated using an Aerne Analytic CL-230D heating block (Aerne Analytic e.K., Weißenhorn, Germany). One syringe was heated to 50 °C and the other to 55 °C for 15–20 min. These syringes were then used to print using the Pharma Printer 1 (software version 3.0.33, CurifyLabs, Helsinki, Finland), and the flow of the material was observed during printing.

The initial printing of the GAB-loaded batches involved inserting one syringe at a time into the printer’s syringe holder. Each syringe was allowed to equilibrate for 15 min inside the printer’s built-in heating element to the designated printing temperature before use. Before printing, excess air was gently expelled by pressing the plunger until the first drop of ink appeared, ensuring consistent extrusion. Two clinically relevant doses, 20 mg and 50 mg, were selected for this study. During the initial print run, Formulation II was used to produce 25 tablets per batch, with six 20 mg and 19 50 mg tablets. Using Formulation I, five 20 mg and nine 50 mg tablets were printed per batch. The placebo batch was used to print 25 tablets of matching weights. After printing, all tablets were placed in a refrigerator at 3–4 °C for 5 min to solidify. Once solidified, they were sealed by using the Roll-E-ZY Press-Piece “25” and Fil-Form template (MediDose Inc., Ivyland, PA, USA) and stored at room temperature until further testing.

To assess ink reusability, the same syringes were reused after 14 days of refrigerated storage. Each syringe was reheated at 55 °C for 20 min, air was expelled as before, and the printer was prewarmed without the syringe for at least 5 min before insertion. Printing was then repeated using the same parameters. Using Formulation II, six 20 mg and 10 50 mg tablets were produced per batch. The amount produced using Formulation I remained consistent with the initial print.

### 2.4 Differential scanning calorimetry (DSC)

Thermal analysis of the printed tablets was performed using a Mettler Toledo STARe DSC 3 system (METTLER TOLEDO, Greifensee, Switzerland). Approximately 5–10 mg of each tablet sample was prepared by cutting and weighing using a Mettler Toledo XS105 analytical balance (METTLER TOLEDO, Greifensee, Switzerland). The samples were sealed in aluminum crucibles with pin-holed lids. DSC measurements were conducted by heating the samples from 20 °C to 200 °C at a rate of 10 °C/min under a nitrogen atmosphere with a flow rate of 50 mL/min, following the procedure described by Sjöholm et al. (13). The resulting thermograms were analyzed using STARe software (version 16.40a, METTLER TOLEDO, Greifensee, Switzerland) to determine the onset and peak temperatures of thermal transitions. Measurements were performed using 50 mg tablets from each batch and printing time point to assess potential variations between batches. In addition to the printed formulations, pure API and placebo samples were also analyzed to serve as references for thermal behavior.

### 2.5 Attenuated total reflectance Fourier transform infrared spectroscopy (ATR-FTIR)

The ATR-FTIR measurements were conducted by choosing one 50 mg tablet from each formulation and printing time. The samples were prepared by cutting a small piece from the bottom of each sample. Dry samples were also prepared by vacuum-drying them overnight at ambient temperature. Each tablet was tested twice from different locations using a Bruker Tensor 27 spectrometer (Bruker Optic GmbH, Ettlingen, Germany) equipped with a Bruker Platinum A225/Q ATR attachment with a diamond crystal (Bruker Optic GmbH, Ettlingen, Germany). The spectra were recorded from 400 to 4,000 cm^−1^ with a resolution of 2 cm^−1^ and 32 scans at ambient temperature using the OPUS software (version 7.0.129, Bruker Optik GmbH, Ettlingen, Germany). The API was also tested, but no sample preparation was needed. The transmittance data were normalized using the following equation:

$$x^{\prime }=\frac{\left(x-\min\;(X)\right)}{\left(\max\;(X)-\min\;(X)\right)}$$

where x is the single data point of the set, min (X) is the minimum value of the dataset, and max (X) is the maximum value of the dataset.

### 2.6 Characterization tests

#### 2.6.1 Morphology

The diameter of the tablets was measured using a standard ruler. The wet mass of each tablet was initially recorded using the printer’s built-in KERN PEJ 620 scale (Kern, Balingen, Germany). Before HPLC analysis, the tablets were reweighed using a Mettler Toledo XP205 analytical balance (Mettler Toledo, Greifensee, Switzerland) to assess potential weight loss during storage. For each batch, the masses of eight tablets were measured, four from each dose group. Additionally, to evaluate potential mass loss of the ink during storage, one GAB-containing syringe was selected. Its mass was measured immediately after the initial printing and again before reprinting using a Mettler Toledo PB303-S balance (Mettler Toledo, Greifensee, Switzerland).

#### 2.6.2 Scanning electron microscopy (SEM)

Surface morphology of the printed tablets was examined using a Zeiss Merlin field emission scanning electron microscope (Zeiss, Oberkochen, Germany). Imaging was performed using secondary electrons at acceleration voltages ranging from 2 to 10 kV and a probe current of 80 pA. The working distance varied between 8.2 and 10 mm, and multiple magnifications were used to capture detailed surface features. For SEM analysis, 50 mg tablets were selected from both Formulation I and II across both initial and reprint time points, excluding the reprinted batches of Formulation I. Additionally, one placebo tablet from each printing time was included. The samples were prepared by slicing a thin section from the bottom of each tablet using a razor blade. These slices were placed in a petri dish and vacuum-dried overnight at ambient temperature using an Across International VO-16020Y vacuum oven (Across International, Livingston, NJ, USA). After drying, the samples were mounted on aluminum stubs using conductive carbon tape. To minimize charging during imaging, aluminum tape was also applied to all samples. Selected samples were sputter-coated with an approximately 20 nm layer of palladium/gold (Pd/Au) before imaging. The pure API was also imaged, but without extensive sample preparation.

#### 2.6.3 Drug content and pH of the tablets

Drug content was quantified using HPLC. From each batch, four tablets of both 20 mg and 50 mg doses were sampled from both initial and reprint time points. For sample preparation, 20 mg tablets were placed in 50 mL volumetric flasks, and 50 mg tablets in 100 mL flasks. Each flask was partially filled with a phosphate buffer (pH 6.9), which was prepared by dissolving 1.2 g of monobasic potassium phosphate in 1 L of HPLC-grade water and adjusting the pH to 6.9 using 5 N potassium hydroxide, and measured with a Metrohm 744 pH meter (Metrohm, AG, Herisau, Switzerland). These flasks were then placed in a water bath maintained at 54–56 °C. The flasks were gently shaken until complete dissolution of the tablets, then allowed to cool to room temperature before being filled to volume. Aliquots were withdrawn and filtered through built-in 0.45 μm filters in HPLC vials.

Quantification was performed using an Agilent 1260 Infinity II LC System (Agilent Technologies, Waldbronn, Germany), equipped with a diode array detector, quaternary pump, multicolumn thermostat, and an autosampler. Data acquisition and processing were carried out using Agilent 2D-LC software (version A.01.04). Chromatographic separation was achieved on an Agilent ZORBAX Eclipse XDB-C18 column (4.6 × 250 mm, 5 μm particle size) using an isocratic method with a mobile phase composed of phosphate buffer (pH 6.9) and acetonitrile in a 94:6 ratio. The solution was vacuum filtered through a 0.45 μm nylon filter before use. The flow rate was set at 0.7 mL/min, with the column temperature maintained at 40 °C. The injection volume was 10 μL, and the run time for each sample was 12 min. Detection was performed at 210 nm with a bandwidth of 4 nm.

The diluent used for sample and stock solution preparation was identical to the mobile phase, but without acetonitrile. A 1 mg/mL GAB stock solution was freshly prepared for each run by dissolving 100 mg of GAB in 100 mL of diluent, using a Mettler Toledo XP205 analytical balance (Mettler Toledo, Greifensee, Switzerland). The solution was sonicated for 5 min using a Branson 3,510 sonicator (Branson Ultrasonics, Danbury, CT, USA) to ensure complete dissolution. Six calibration standards (0.1–0.6 mg/mL) were prepared. Method parameters were selected based on previously published papers (14, 15) and by using an already existing method shared by CurifyLabs.

The pH of the tablets was measured from both the initial prints and the reprinted batches. One 50 mg sample was taken from each batch, weighed using a Mettler Toledo XP205 analytical balance, and transferred into a 100 mL volumetric flask. The samples were dissolved in deionized water and heated in a 55 ± 1 °C water bath until fully dissolved. After cooling to room temperature, the flasks were filled to volume, and the solutions were transferred into beakers. The pH was recorded using a VWR PH 20 pH-meter (VWR International, Radnor, PA, United States). In addition, the pH of one placebo tablet was measured following the same procedure to serve as a reference.

### 2.7 Statistical analysis

All statistical analyses were performed using JASP software (version 0.95.4.0, JASP Team, Amsterdam, The Netherlands). A one-way analysis of variance (ANOVA) was used to ensure that there were no statistically significant differences in content between batches. To evaluate the assumption of homogeneity of variances, Levene’s test was applied. If this assumption was violated (*p* < 0.05), Welch’s ANOVA was used as a more appropriate alternative. In cases where no statistically significant differences were found between batches, independent samples t-tests were conducted to determine whether the content differed significantly between initial and reprint time points, with a significance threshold set at *p* = 0.05.

## 3 Results and discussion

Initial reusability testing demonstrated that the refrigerated placebo formulation could be successfully reprinted after heating. At 50 °C, 20 min of heating was required to achieve printable viscosity, while at 55 °C, extrusion was successful after both 15 and 20 min. The best printability was observed at 55 °C after 20 min, making it the preferred condition for this experiment. Contrary to expectations, the higher temperature did not compromise the structural integrity of the printed tablets. This test suggested that the formulation retained its rheological properties and could be printed without modifying printer settings.

The initial printing of Formulation II yielded high-quality tablets with accurate wet mass and no visible undissolved API, most likely due to GAB having a high water solubility of >100 mg/mL (16). However, after 4 days of storage, solid white, crystal-like structures appeared in nearly all tablets, particularly at the bottom of the blisters, and similar crystallization was observed in the corresponding syringes, accompanied by large air pockets. Brown particles, likely originating from water-insoluble excipients, were also present in both the tablets and syringes. In contrast, Formulation I and the placebo showed no changes, indicating that the higher GAB concentration in Formulation II likely exceeded the solubility threshold during storage, leading to nucleation and crystal growth as water evaporated, a phenomenon previously reported in GAB-containing formulations (13, 17). The improved stability of Formulation I may also be attributed to a higher PS80-to-GAB ratio, though further investigation is needed.

**Figure 1:**
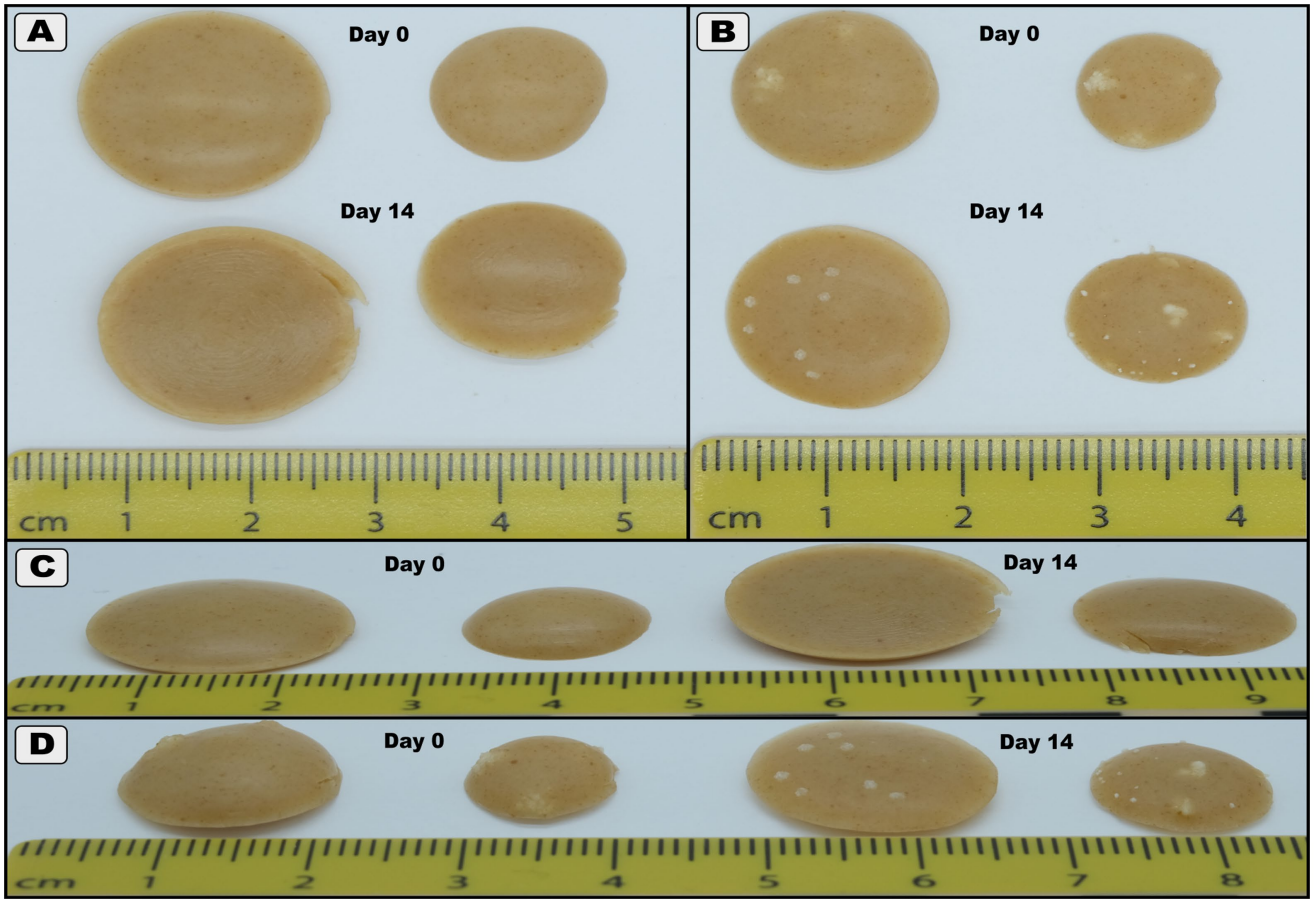
Images of the tablets from the initial printing (Day 0) and the reprinting (Day 14). The larger tablets are 50 mg, and the smaller ones next to them are 20 mg. **(A)** Tablets with Formulation I pictured from above, **(B)** Tablets with Formulation II pictured from above, **(C)** Tablets with Formulation I pictured from the side, **(D)** Tablets with Formulation II pictured from the side.

When reprinting Formulation I after 14 days of refrigerated storage, we observed that a slight amount of manual extrusion was required to initiate flow before placing the syringe in the printer. This initial resistance aligns with previous reports of increased extrusion pressure in stored semi-solid formulations, likely due to reduced inter-particle lubrication from water loss and possible depletion of silicone oil within the syringe, potentially intensified by the presence of PS80 (10, 18, 19). Reheating Formulation II revealed persistent crystalline structures within the walls of the syringes, reinforcing the hypothesis of GAB supersaturation as the cause of nucleation. Printing with this formulation resulted in nozzle clogging, inconsistent wet mass, and incomplete prints that are commonly associated with high solid content in extrusion systems (20). Post-print inspection revealed crystal clusters at the bottom of the syringe, likely dislodged during extrusion. Additionally, reprinted tablets, particularly the 50 mg target dose units from Formulation I, exhibited increased diameter and slight base curvature, likely due to ink spreading at elevated temperatures (Figure 1) (21). These tablets were also more fragile, with thinner edges prone to damage during handling, suggesting that future studies should consider optimizing blister design to better support the formulation during solidification.

Mass uniformity was assessed by comparing the wet masses of tablets from both initial and reprinted batches. Formulation I demonstrated consistent tablet weights across all prints, with no trends in variation between initial and reprinted samples, as also reflected in the overlapping standard deviations (Table 1). In contrast, while the initial prints of Formulation II showed acceptable mass consistency, the reprinted tablets exhibited significant deviations, particularly in the 500 mg target group, likely due to nozzle blockages during printing, which also led to the exclusion of some tablets from analysis. To evaluate the impact of storage, water loss was quantified across all formulations, ranging from 2.04 to 6.34%, with Formulation II showing a larger average, but also greater variability. This mass loss may have begun during the solidification of the tablets and continued during storage, which could have been prevented with a blister with hermetic sealing. A 230 mg reduction in mass was also recorded in a single Formulation II syringe over the storage period, supporting the hypothesis that water evaporation contributed to the crystallization observed in the ink. Compliance with the European Pharmacopeia 2.9.5 guideline for uniformity of mass (22) was assessed using a reduced sample size. For Formulation I tablets, a 5% deviation threshold was applied. For Formulation II, a 7.5% deviation was used for the 200 mg tablets and 5% for the larger 500 mg tablets, in accordance with the guideline. All batches of Formulation I met the specified limits, whereas Formulation II failed to comply. Among the initial prints, one 200 mg tablet out of 12 exceeded the allowable deviation, and nearly all reprinted tablets fell outside the acceptable range. These findings highlight the instability of the higher-concentration formulation during storage and its adverse impact on print reliability and dose accuracy.

**Table 1:** Average wet masses and calculated loss of mass.

Formulation	Target weight (mg)	Wet mass initial prints (mg)	Loss of mass (%)	Wet mass reprints (mg)	Loss of mass (%)
Formulation I	400	413.8 ± 5.8	3.1 ± 0.4	410.3 ± 6.7	3.3 ± 0.6
	1,000	1,006.8 ± 13.4	2.0 ± 0.3	1,006.7 ± 13.4	2.3 ± 0.4
Formulation II	200	214.8 ± 9.0	5.0 ± 1.0	^1^227.9 ± 21.0	^1^4.7 ± 5.9
	500	503.2 ± 9.8	2.8 ± 1.3	^2^436.3 ± 196.6	^2^6.3 ± 7.6

Values are shown as the mean ± SD and loss of mass as a percentage (*n* = 12) ^1^(*n* = 7) ^2^(*n* = 9).

**Figure 2:**
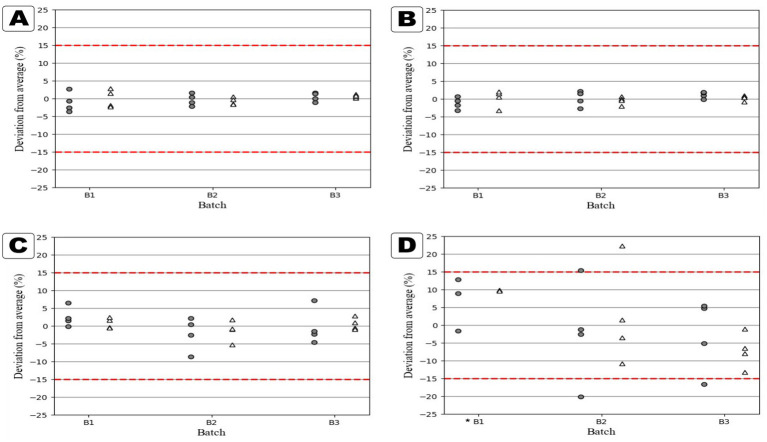
Graphs showing the content deviations from the average dose as a percentage. The round gray shapes are 20 mg tablets, and the white triangular shapes are 50 mg tablets. The red lines have been added to show the limits described in Ph.Eur 2.9.6. **(A)** Formulation I initial prints, **(B)** Formulation I reprints, **(C)** Formulation II initial prints, **(D)** Formulation II reprints (*n* = 8) *(*n* = 5).

Content uniformity analysis revealed distinct differences between the two formulations. Tablets produced with Formulation I showed consistent drug content across both initial and reprinted batches (Table 2). These tablets showed no statistically significant difference in content between the initial and reprint time points (*p* > 0.05), indicating that storage did not affect the formulation. All Formulation I tablets also met the European Pharmacopeia 2.9.6 criteria for uniformity of content (23), i.e., less than 15% deviation, even though a larger sample size was used, confirming the reliability of this formulation for on-demand printing (Figure 2). Formulation II initially performed well, with acceptable content uniformity in both dose groups. However, the reprinted tablets showed significant deviations, with 20 mg tablets averaging 17.25 mg of GAB and 50 mg tablets averaging 35.86 mg, both falling outside acceptable limits. Four tablets from the reprinted batches failed the pharmacopeial criteria, indicating poor reproducibility. These deviations are consistent with the earlier observations of crystallization and nozzle clogging, which likely disrupted ink homogeneity and led to uneven drug distribution.

**Table 2:** The target dose and average drug content.

Formulation	Target dose (mg)	Drug content initial prints (mg)	Drug content reprints (mg)
Formulation I	20	21.52 ± 0.37	21.58 ± 0.39
	50	52.38 ± 0.82	52.63 ± 0.78
Formulation II	20	21.86 ± 0.97	^1^17.25 ± 1.93
	50	51.59 ± 1.12	^2^35.86 ± 3.97

The content is shown as the mean ± SD (*n* = 12) ^1^(*n* = 11) ^2^(*n* = 10).

The pH of the tablet dispersions was measured from both the initial printed samples and the reprinted batches to assess any changes related to the storage of the ink. For the 5% (w/w) formulation, the average pH was 5.07 ± 0.06 in the initial prints and 5.04 ± 0.03 in the reprints. The 10% (w/w) formulation showed a slightly higher pH of 5.18 ± 0.06 initially, which decreased to 5.10 ± 0.07 in the reprinted samples. Although no statistical analysis was performed, the values remained consistently around pH 5 across all batches, with no clear trend of increase or decrease. For reference, the pH of the placebo tablet was measured at 4.85, indicating that the presence of GAB slightly increases the pH of the formulation toward a more neutral range. This is relevant for GAB, which has been shown to have a slower degradation rate in mildly acidic and neutral environments, where it exists mostly as a zwitterion, due to its pKa values (10.7 and 3.7) (14, 24). These consistent pH values across initial and reprinted batches, remaining close to the optimal range for the GAB’s chemical stability, further support the integrity of the formulations and their suitability for reuse.

**Figure 3:**
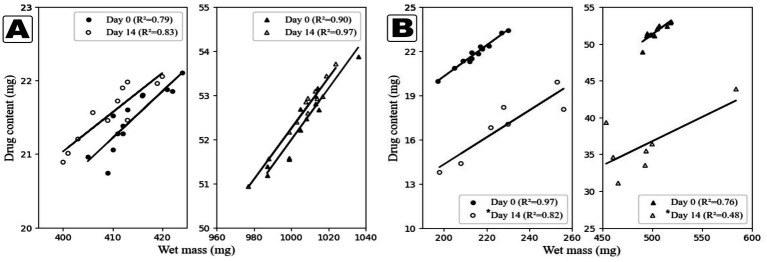
Correlation between drug content and wet mass with both formulations and doses plotted individually. **(A)** Formulation I tablets, **(B)** Formulation II tablets (*n* = 12) *(*n* = 7).

In this study, the wet mass-to-content graph was employed to compare different printing times, enabling a visual assessment of whether variations in drug content could be detected among tablets of identical mass. This approach also facilitated the evaluation of batch homogeneity. A distinct linear correlation was observed between tablet mass and drug content, most notably in Formulation I, where the 50 mg target dose tablets exhibited the strongest relationship (Figure 3). These findings reinforce the utility of wet mass as a reliable indicator of dose accuracy in SSE-printed formulations, aligning with conclusions drawn in a previous publication by Johannesson et al. (25). The reprinted tablets from Formulation I showed improved correlation, and a minor increase in concentration was observed visually, which is most likely caused by the slight water evaporation during storage. Formulation II initially exhibited a good mass-to-content correlation, especially in the lower-dose tablets. However, the reprinted 50 mg tablets showed poor correlation, indicating that the ink had become non-homogeneous after storage. While crystal formation inside the syringes during storage likely contributed to this variability, the fact that these structures did not significantly affect content measurements suggests they may have been composed primarily of excipients rather than API. This may indicate that the detected crystals were most likely co-crystals formed within the syringe.

**Figure 4:**
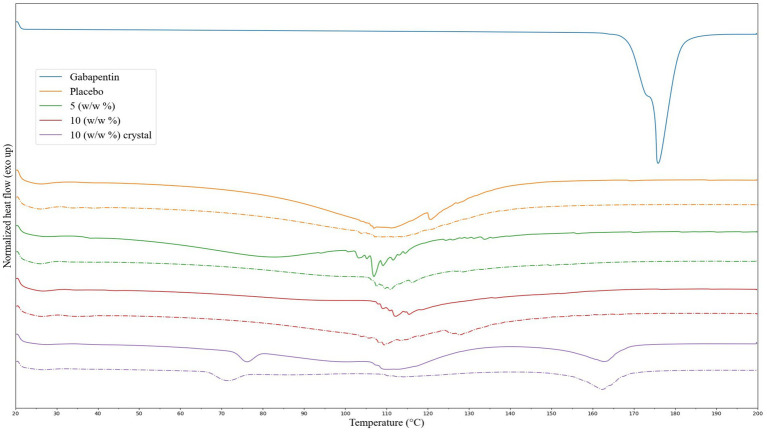
DSC thermograms of GAB, placebo, and both formulations. The dotted lines represent the reprints.

Differential scanning calorimetry (DSC) was used to investigate the thermal behavior of the printed tablets, placebo, and pure GAB. The pure API exhibited an onset at 167 °C and an endothermic peak at 176 °C, consistent with previously reported values for anhydrous GAB (Figure 4) (6, 26). These peaks were absent in the printed tablets, likely due to the API being dissolved within the gelatin-based matrix (27). All tablet samples also displayed a broad endothermic valley between 40 °C and 110 °C, attributed to moisture release from the hydrated gelatin (28). Additional peaks were observed within this range, including a distinct peak at 120 °C in the placebo, likely corresponding to the flash point of PS80 (26). In GAB-containing tablets, this peak appeared slightly lower, around 115 °C, and was more pronounced in the chewable tablets, possibly indicating hydrogen bonding between GAB and excipients, which may have enhanced the visibility of thermal transitions when disrupted (29). The reprinted tablets showed similar thermal profiles with minor shifts in peak positions, suggesting no major changes in formulation behavior after storage.

DSC analysis of the crystalline structures observed in Formulation II tablets revealed two distinct endothermic peaks: one at 77 °C and another at 164 °C, with corresponding onsets at 72 °C and 148 °C (Figure 4). In the reprinted samples, these onset values shifted slightly to 76 °C and 153 °C, with endothermic events at 72 °C and 162 °C. The lower peak may correspond to the dehydration of a GAB monohydrate form, while the higher peak likely represents the melting of GAB at a lower temperature compared to the pure drug (30). This lower melting point has been observed before for the co-crystal form of the API. Additional peaks around 110 °C were attributed to excipient residues introduced during sample preparation. Notably, no peaks were observed in the 87–91 °C range, which would indicate the presence of GAB lactam, a known degradation product (13). These findings support the hypothesis that the crystalline structures in Formulation II were related to GAB co-crystallization with one or more excipients, likely triggered by water loss and supersaturation during storage.

**Figure 5:**
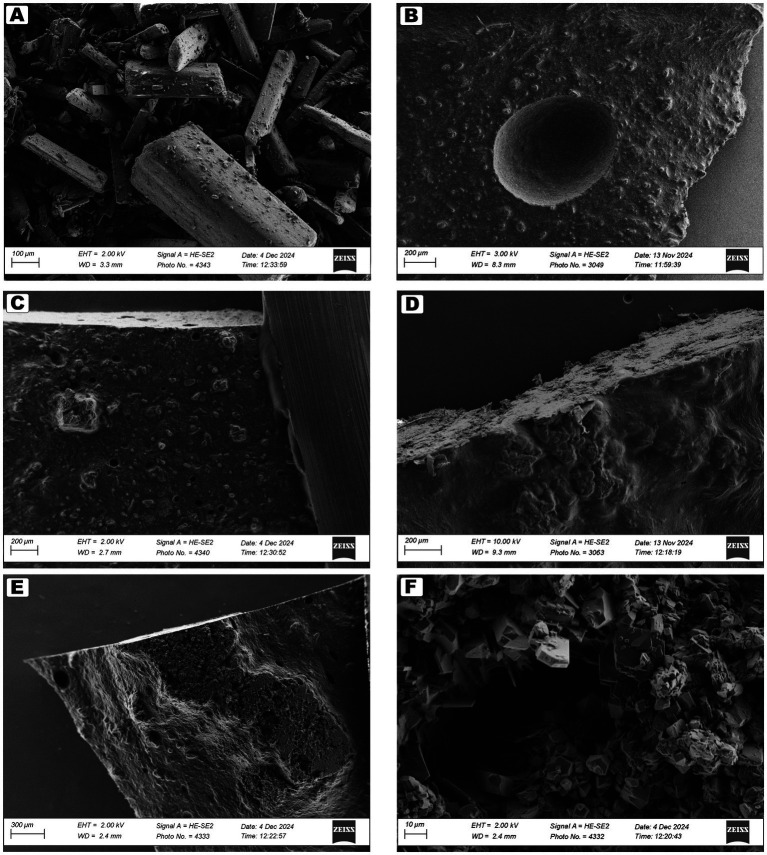
SEM images of the samples. The placebo and Formulation II are coated. **(A)** GAB, **(B)** Placebo, **(C)** Formulation I, **(D)** Formulation II, **(E)** Formulation II reprint, **(F)** Formulation II reprint with a higher magnification.

Scanning electron microscopy (SEM) was employed to investigate the surface morphology of the printed tablets and to further examine the white structures observed in the samples printed with Formulation II. The pure anhydrous GAB powder exhibited characteristic needle-like crystals ranging from under 100 μm to over 700 μm, consistent with previous reports (Figure 5) (30, 31). Compared to the placebo, Formulation I tablets showed slightly rougher surfaces, with a distinct structure approximately 200 μm in diameter, likely resulting from partial recrystallization of GAB during vacuum drying, which has also been seen in other SSE formulations (27, 32). All samples, including the placebo, displayed small holes in their structures, attributed to moisture loss during the drying process. In contrast, Formulation II tablets, and particularly those from the reprinted batches, exhibited significantly rougher surfaces in regions where white changes had been observed macroscopically. High-magnification imaging revealed crystal-like features with a plate-like morphology, some measuring less than 10 μm. These structures differed from the characteristic spiked morphology of anhydrous GAB and were more consistent with the monohydrate form or cocrystals of GAB with excipients (30, 31). The presence of these crystal structures supports the hypothesis that recrystallization occurred during storage and was likely driven by water evaporation and supersaturation within the ink, creating cocrystals. These findings align with the DSC results and highlight the importance of avoiding supersaturation during storage.

**Figure 6:**
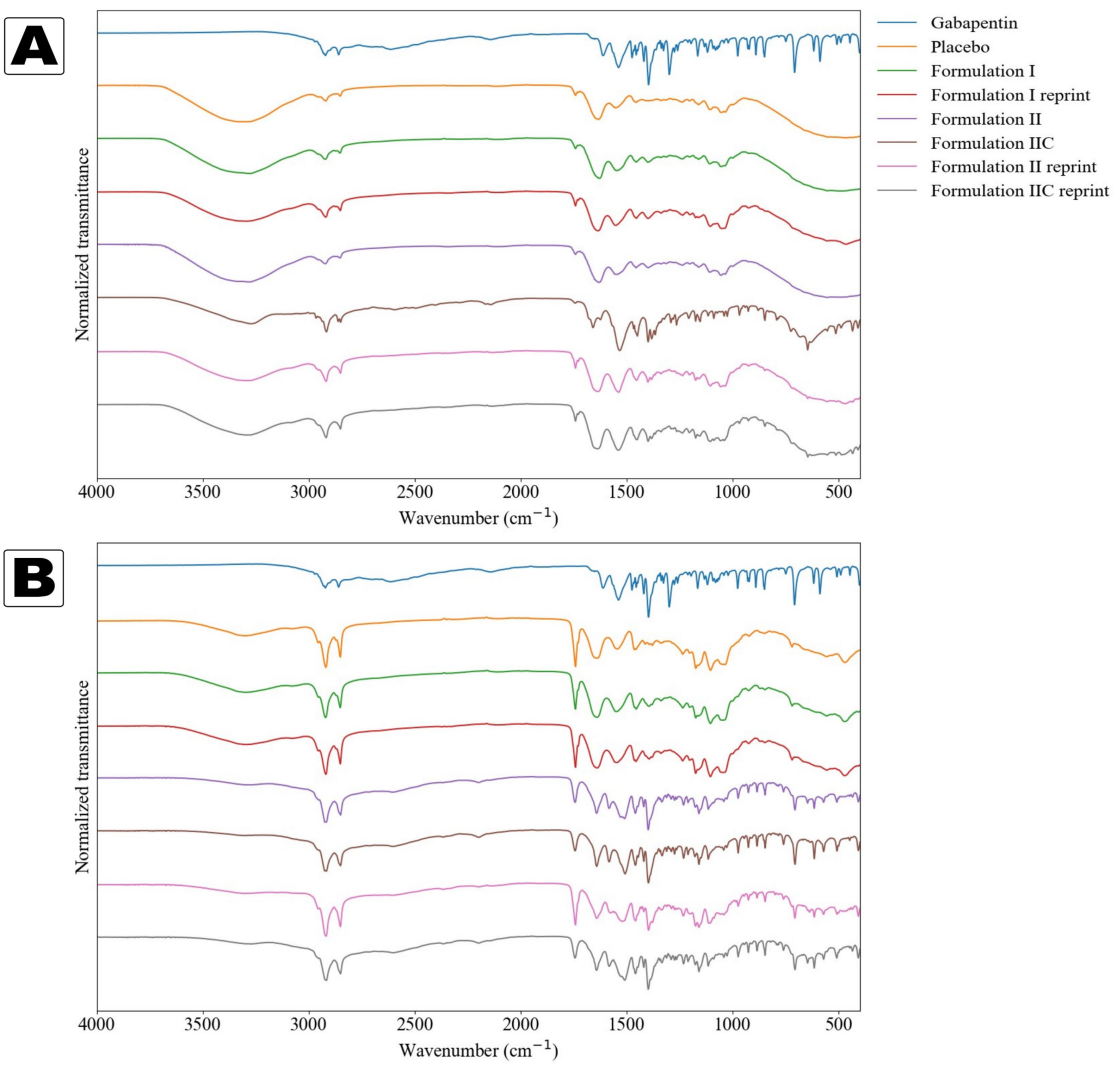
ATR-FTIR spectra of pure GAB and the samples before and after drying. The cut-out crystals from Formulation II samples are marked with a C. **(A)** Samples without drying, **(B)** Samples after drying.

ATR-FTIR spectroscopy was employed to investigate spectral differences between placebo and GAB-loaded formulations. This comparative analysis was further used to assess whether the observed structures exhibited characteristic peaks associated with GAB, thereby supporting the hypothesis of GAB crystallization within the printed tablets. Pure GAB exhibited characteristic peaks, including N–H stretching at 1,615 cm^−1^ and a minor C=O stretching peak at 1,652 cm^−1^, attributed to the carboxylic acid group, consistent with previous reports (Figure 6) (30). As a zwitterionic compound, GAB showed no absorption in the 3,300–3,500 cm^−1^ region, while NH^3+^ stretching vibrations were observed at 2,921 cm^−1^ and 2,859 cm^−1^ (26). A peak at 2,140 cm^−1^ was assigned to C–N or side chain vibrations (33). No spectral differences were observed between different measurement locations, so a representative spectrum was selected for analysis.

In both the wet placebo and GAB-containing samples, a broad O–H stretching peak appeared at 3,279 cm^−1^, while a peak at 1,740 cm^−1^, also present in the placebo and crystal samples, suggested the presence of excipients or possible cocrystal formation with GAB, as supported by DSC and SEM data (Figure 6) (34). A distinct peak at 1,658 cm^−1^ was observed in the wet Formulation II crystal sample, potentially indicating the monohydrate form I of GAB, though this was not consistently seen across other samples (30). The dried samples retained the broad O–H peak, with Formulation I showing the most prominent signal. The presence of GAB-specific peaks in the crystal spectra confirmed the incorporation of the API, and the enhanced visibility of these peaks in dried Formulation II samples suggested recrystallization during the drying process, corroborated by the SEM and DSC observations.

## 4 Conclusion

This study demonstrated the successful reusability of water-based gelatin inks for 3D printing chewable veterinary Gabapentin tablets using semi-solid extrusion after 2 weeks of refrigerated storage. It is the first to evaluate a gelatin-based ink specifically designed for veterinary patients for reusability. The optimized low-concentration formulation consistently produced tablets with acceptable mass and content uniformity, while the higher-concentration formulation showed instability due to water evaporation, leading to API supersaturation, crystal formation, and compromised print quality. Characterization using HPLC, DSC, SEM, and FTIR confirmed drug incorporation, formulation homogeneity, and indications of cocrystal formation. To further strengthen the understanding of these systems, future studies should incorporate X-ray powder diffraction to clarify the nature of co-crystallization and evaluate microbiological stability, mechanical strength, disintegration behavior, long-term storage effects, and *in vivo* palatability and acceptability testing. These aspects are essential for establishing the clinical relevance and robustness of reusable ink formulations in veterinary medicine.

The results support sustainable, on-demand manufacturing of personalized veterinary medicines, particularly in clinical settings where individualized dosing and small-batch production are essential. Reusable ink systems could streamline compounding workflows, reduce waste, and provide more practical, palatable, and patient-friendly formulations, ultimately improving compliance and animal welfare. This is especially valuable for chronic treatments such as Gabapentin administration in canine neuropathic pain, where frequent dose adjustments are needed. By demonstrating the feasibility of reusing gelatin-based inks, this study not only addresses sustainability and clinical applicability but also highlights a pathway toward regulatory frameworks that can enable broader clinical adoption of 3D printed veterinary medicines.

## Data Availability

The original contributions presented in the study are included in the article/supplementary material, further inquiries can be directed to the corresponding author.
